# Clinical Phenotype and Inheritance in Patients With C9ORF72 Hexanucleotide Repeat Expansion: Results From a Large French Cohort

**DOI:** 10.3389/fnins.2020.00316

**Published:** 2020-04-28

**Authors:** Florence Esselin, Kevin Mouzat, Anne Polge, Raul Juntas-Morales, Nicolas Pageot, Elisa De la Cruz, Emilien Bernard, Emmeline Lagrange, Véronique Danel, Sébastien Alphandery, Laura Labar, Erika Nogué, Marie-Christine Picot, Serge Lumbroso, William Camu

**Affiliations:** ^1^Centre de référence SLA, CHU Gui de Chauliac, Montpellier, France; ^2^Laboratoire de Biochimie et Biologie Moléculaire, CHU Nimes, Univ. Montpellier, Nimes, France; ^3^Département de Neurologie, Centre SLA, CHU Wertheimer, Lyon, France; ^4^EFSN, CHU Grenoble Alpes, Grenoble, France; ^5^Centre de référence SLA, CHU Salengro, Lille, France; ^6^CHU Montpellier, Unité de Recherche Clinique et Epidémiologie (Département Information Médicale), INSERM, Montpellier, France; ^7^CHU Montpellier, Unité de Recherche Clinique et Epidémiologie (Département Information Médicale), INSERM, Centre d’Investigation Clinique 1411, Univ Montpellier, Montpellier, France

**Keywords:** ALS, C9ORF72, clinical phenotype, familial, cohort

## Abstract

**Background:**

In familial amyotrophic lateral sclerosis (ALS) cases, the presence of an abnormal C9ORF72 repeat expansion (C9RE) is the most frequent genetic cause identified. Various clinical phenotypes have been described in relation to the presence of C9RE, including psychiatric disorders or Huntington-like symptoms. In a subset of sporadic ALS, C9RE has also been described. In the present study, all index cases with ALS and C9RE identified in our center and their clinical profile, as well as neurological and psychiatric characteristics of identified family members, were described. Clinical characteristics of ALS patients were compared to 999 patients with sporadic ALS (SALS) from our database.

**Results:**

From the 70 index cases with ALS identified, a total of 200 individuals were studied, 118 with ALS, 32 with fronto-temporal lobe degeneration (FTD), 37 with ALS/FTD, and 13 with psychiatric disorders. A familial history was present in 57 of the index cases (81%). In ALS and ALS/FTD cases with C9RE, the age of onset (AoO) was earlier than that in SALS cases, *p* < 0.0001 and *p* = 0.008, respectively. Sporadic cases with C9REALS (*n* = 13) had an earlier AoO compared to familial C9REALS ones, *p* < 0.0001. Within families, there was an earlier AoO in index cases and their siblings compared to their parental generation (*p* < 0.01). There was also a significant intrafamilial correlation for bulbar onset of ALS. The parental generation had significant female predominance compared to index cases and their siblings (sex ratio 0.47 vs. 1.4, *p* = 0.004), and this predominance was also present when considering parent–child pairs. In the group with psychiatric disorders, suicide was prominent (*n* = 9) and mean age was 54 years.

**Conclusion:**

Although our sample size is rather limited, the earlier AoO in index cases and their siblings compared to the parental generation may suggest an anticipation. Reasons for predominance of female transmission are unclear, but the hypothesis that gender influences transmission of the genetic trait or C9RE size variation may be taken into account. Intrafamilial correlation suggests that genetic aspects underlie the occurrence of bulbar onset in ALS patients. Studies on larger samples are warranted to confirm those results.

## Introduction

Amyotrophic lateral sclerosis (ALS) is a neurodegenerative disorder characterized by degeneration of upper and lower motoneurons, leading to death in a median time of 3 years after onset (Hardiman et al., 2011). The disease is inherited in 5–10% of the cases, and several causal mutations have been described (Volk et al., 2018). The most frequent one is the C9ORF72 repeat expansion (C9RE) that accounts for 30–50% of the familial cases in European and American countries, while the expansion is rare in Asia (DeJesus-Hernandez et al., 2011; Renton et al., 2011; Majounie et al., 2012). This expansion is now also recognized as the most frequent cause of familial fronto-temporal lobe degeneration (FTD). The clinical spectrum of C9RE cases may also extend to ALS/FTD and potentially to atypical parkinsonism, Huntington-like disease, or psychiatric disorders (Byrne et al., 2013; Cooper-Knock et al., 2014). The frequency as well as the clinical profile of ALS with the C9RE seem to be rather homogeneous, with a predominant bulbar onset, an age of onset of 58 years, and a mean disease duration shorter than for sporadic cases (Majounie et al., 2012; Snowden et al., 2013; Cooper-Knock et al., 2014; Umoh et al., 2016; Trojsi et al., 2019). Conversely, the other phenotypes associated with C9RE are not well defined and some remain in dispute (Ticozzi et al., 2014; Solje et al., 2016; Devenney et al., 2018; Martins et al., 2018; Sellami et al., 2019; Silverman et al., 2019). The proportion of ALS/FTD varies from 6 to 50%, reports of atypical phenotypes are scarce, and physicians are lacking a systematic study to help clarify the exact spectrum linked to C9RE. The difficulty is reinforced by the existence of C9RE in ∼3% of apparently sporadic cases and by an incomplete and age-related penetrance in both familial and sporadic cases (Majounie et al., 2012; Murphy et al., 2017; Volk et al., 2018). Incomplete familial enquiry might lead to potential underestimation of familial cases, as well as the existence of phenotypes not yet recognized as part of the disease spectrum, and difficulties asserting diagnosis. Improving knowledge in the phenotype of patients with C9RE is of clinical importance all the more as future gene therapies are being developed for treating patients. We thus undertook a systematic phenotypic study of all ALS families recruited in our ALS clinic, and thoroughly described phenotypes of all family members. Our secondary objective was to compare intra- and interfamilial phenotype variability.

## Methods

### Patients

All patients and families were identified at the ALS clinic of Montpellier, France. As early as 1996, all FALS cases were collected and patients systematically followed quarterly. Between 1996 and 2012, blood collection was part of a DNA banking stored at Genethon, and all the patients gave written consent for participating to the genetic research. This research had been approved by the ethics committee of Pitié-Salpêtrière hospital. Since 2012, with the description of C9RE, genetic analysis of ALS cases was part of the diagnostic procedure, and all subjects also gave informed consent for genetic testing.

### Phenotype Characterization

Clinical profiles of index cases and affected family members have been collected. For each patient, the following information was collected: age at onset, gender, site of onset, ALS functional rating scale–revised (ALSFRS-R) score, disease duration, and presence of dementia (Kimura et al., 2006). Familial inquiry considered relatives with ALS, ALS/FTD, FTD, psychiatric disorder, parkinsonism, and any other potential neurodegenerative disorder. Age at onset, disease duration, and/or age at death were collected for each family member identified, and neurologists as well as psychiatrists who followed a family member were contacted for diagnosis ascertainment.

### Genetic Testing

Between 1996 and 2018, all index cases and other family members for whom blood collection was possible were studied for the presence of an abnormal C9RE by the Biochemistry and Molecular Biology department of CHU de Nîmes, as already described (Akimoto et al., 2014). All index cases were also checked for mutations in the main genes responsible for familial ALS: SOD1, TARDBP, and FUS genes. The study of these genes did not show any additional mutation.

### Statistical Analysis

The patients’ characteristics were reported and compared with data from sporadic ALS patients recorded in our prospective database (*n* = 999). Quantitative variables (age at onset, age at death, disease duration, ALSFRS-R score) were expressed by their mean ± standard deviation, and the qualitative variable (gender, site of onset) with frequencies and proportions (%) of the different categories.

Comparisons between groups for age of onset, age at death, disease duration, and ALSFRS-R score were done using Student’s *t*-test. Gender and sites of onset were compared between groups using chi-squared test. The type I error rate was 0.05. Statistical analysis was performed using XLSTAT statistical and data analysis solution (Addinsoft, Paris, France)^1^.

Phenotype similarities within families were expressed by the intracluster correlation coefficient (ICC), which compares the within-group variance to the between-group variance (Killip et al., 2004). Mathematically, it was the between-cluster variability divided by the sum of the within-cluster and between-cluster variabilities. In human studies, it is usually small. The 95% confidence intervals (CIs) of each ICC will be estimated by bootstrap with resampling including the delivery of 1,000 samples. A negative lower bound will be truncated to zero. The ICC associated with 95% CI containing the value 0 will be considered as not significantly different from 0. These tests were performed using SAS enterprise guide 7.13.

## Results

Between 1996 and 2018, we identified 70 individuals with ALS referred to our center who were shown to carry abnormal C9RE (index-cases). A familial history of ALS or FTD or both was identified in 57 of them; one family originated from North Africa (Tunisia), with all the others being of European descent. The pedigree study identified a total of 200 individuals with ALS (*n* = 118), FTD (*n* = 32), ALS/FTD (*n* = 37), as well as psychiatric disorders (*n* = 13).

### Phenotype of Families

Families with only ALS cases and families with coexistence of ALS cases and FTD cases were the most represented, accounting for 58% of the 57 families (Table 1). The number of cases per family was higher in those with a coexistence of the three types of neurodegenerative disorders (4.5) and in families with ALS and ALS/FTD cases (3.4), compared to 2 and 2.6 for ALS/FTD- and ALS-only families, respectively. Psychiatric disorders were noted in almost all of the family types.

**TABLE 1:** Phenotype of families.

	ALS only	ALS/FTD only	ALS and FTD	ALS and ALS/FTD	ALS/FTD and FTD	ALS and ALS/FTD and FTD
Families (n)	19	3	14	10	5	6
ALS	50	–	28	19	–	13
ALS/FTD	–	6	–	15	5	7
FTD	–	–	18	–	6	7
Total	50	6	46	34	11	27
Cases per family	2.63	2	3.28	3.4	2.2	4.5
Psychiatric disorders (n)	4	0	2	3	2	1
Disease types	3 suicides 1 schizophrenia		1 bipolar 1 hypochondria	Suicides	1 suicide 1 unknown	Suicide

### Patients’ Characteristics

A total of 106 individuals out of the 200 patients of this cohort could be studied genetically, and the presence of C9RE was confirmed in all of them. The vast majority of the patients with neurological disorders had ALS (*n* = 118, Table 2). Clinical characteristics of these patients had some differences with the 999 sporadic ALS cases from our database, with a female predominance (gender ratio 0.9) compared to 1.25 in SALS, but this was not significant. In ALS patients with C9RE as well as in SALS, upper limb onset was underrepresented and lower limb onset was predominant (43%). Conversely, the age of onset was earlier in C9RE patients than in SALS cases, 57.5 years vs. 65.7 years, which was highly significant (*p* < 0.0001). There was no difference for mean disease duration. Interestingly, except for the gender ratio, ALS/FTD cases were rather similar to ALS patients, and the age of onset was also significantly earlier in ALS/FTD cases with C9RE, *p* = 0.008. When comparison between ALS and ALS/FTD patients was refined according to sites of onset, the only difference was disease duration, which was shorter in ALS/FTD cases (Table 3). On the contrary, FTD cases had a later onset and a much longer disease duration than ALS and ALS/FTD cases. There was no phenotype difference between women and men both in ALS and ALSFTD cases.

**TABLE 2:** Characteristics of the population.

	ALS	ALS/FTD	FTD	Sporadic ALS
n	118	37	32	999
Gender: M/F (ratio)	56/62 (0.9)^*a*^	22/15 (1.46)^*a*^	14/18 (0.78)	555/444 (1.25)
Bulbar onset (%)	39	36.1	–	35.7
UL onset (%)	18	16.6	–	25.0
LL onset (%)	43	41.7	–	39.3
Dementia onset (%)	–	5.6	–	–
Age at onset	57.5 ± 10.4^*b*^	60.4 ± 7.0^*c*^	65.7 ± 9.4	65.7 ± 11.8
*n*	*94*	*34*	*10*	*999*
Age at death	60.7 ± 10.0	63.7 ± 6.7	72.3 ± 8.5	68.8 ± 11.5
*n*	*86*	*30*	*13*	*999*
Disease duration	40.3 ± 24.4^*a*^	30.0 ± 18.2^*a*^	87.6 ± 35.7	37.2 ± 31.7
Median survival	33.5	26.4	75	29.6
*n*	*86*	*29*	*8*	*999*

*M/F, male/female; UL, upper limb; LL, lower limb. ^*a*^Not statistically different vs. sporadic ALS cases. ^*b*^vs. sporadic ALS: p < 0.0001. ^*c*^vs. sporadic ALS: p = 0.008.*

**TABLE 3:** Clinical characteristics of ALS and ALS/FTD patients.

	Age of onset	Age at death	Disease duration	ALSFRS-R score
**ALS**				
Bulbar onset	59.4 ± 10.0(33)	61.1 ± 10.8(30)	37.0 ± 28.7(30)	39.0 ± 3.6(25)
UL onset	56.4 ± 10.7(16)	58.5 ± 9.1(14)	42.1 ± 30.6(14)	35.6 ± 9.5(9)
LL onset	56.3 ± 10.5(35)	59.9 ± 10.7(32)	43.3 ± 19.8(32)	40.0 ± 4.5(24)
**ALS/FTD**				
Bulbar onset	60.0 ± 9.4(12)	63.8 ± 8.8(10)	22.6 ± 9.1(10)	38.5 ± 8.1(11)
UL onset	59.3 ± 5.8(6)	61.4 ± 5.3(5)	39.4 ± 26.4(5)	31.4 ± 16.8(6)
LL onset	61.0 ± 5.9(14)	64.7 ± 6.0(12)	30.7 ± 18.1(12)	36.6 ± 11.0(9)
Dementia onset	61.0 ± 4.8(2)	64.3 ± 7.3(2)	39.8 ± 30.5(2)	
**ALS**				
Men	56.7 ± 10.6(45)	60.0 ± 10.1(38)	35.8 ± 19.0(37)	39.6 ± 5.3(32)
Women	58.0 ± 10.2(48)	60.9 ± 10.1(46)	43.7 ± 27.7(49)	37.9 ± 5.9(26)
**ALS/FTD**				
Men	59.4 ± 6.6(21)	62.5 ± 6.5(18)	30.5 ± 17.6(18)	40.0 ± 6.7(15)
Women	61.9 ± 7.6(13)	65.5 ± 6.9(12)	29.2 ± 19.9(11)	31.8 ± 14.0(11)

*Values are means ± SD (n). ALSFRS-R, amyotrophic lateral sclerosis rating scale-revised; UL, upper limb; LL, lower limb.*

### Apparently Sporadic Cases

A total of 13 patients, 9 with ALS and 4 with ALS/FTD, had no family history of ALS, ALS/FTD, or FTD. For two patients, the pedigree could not be found, and thus it was not possible to conclude precisely about the apparently sporadic nature of their disorder. In three cases, the parents of the patients died before the age of 70 years old, corresponding to a non-informative pedigree. In three patients, both parents were alive with age more than 80 years old. In two cases, one parent died with age between 70 and 80 years old, and the other was alive. In four index cases, psychiatric antecedents were recorded in six persons, including one suicide. Phenotype comparison with cases with familial history was limited due to the size of the samples (Table 4). However, the age of onset was significantly earlier in apparently sporadic ALS cases, 45.5 years vs. 58.8 years (*p* < 0.0001), but not in the ALS/FTD group, which was only composed of four patients. Conversely, in ALS/FTD cases, disease duration was shorter but the group was composed of only three patients.

**TABLE 4:** Clinical characteristics of familial and apparently sporadic cases.

	ALS (*n* = 118)		ALS/FTD (*n* = 37)	
	AS cases	Familial	AS cases	Familial
Gender: M/F (ratio)	4/5 (0.8)	53/56 (0.9)	2/2 (1)	20/13 (1.5)
Bulbar onset (%)	22	41	50	36
UL onset (%)	0	20	25	16
LL onset (%)	78	39	25	42
Dementia onset (%)	–	–	–	6
Age of onset^*a*^	45.5 ± 6.5^*b*^	58.8 ± 9.9	64.6 ± 6.9	59.8 ± 7.0
*n*	*9*	*85*	*4*	*30*
Age at death^*a*^	49.4 ± 7.6	62.0 ± 9.5	65.8 ± 8.3	63.4 ± 6.7
*n*	*9*	*77*	*3*	*27*
Disease duration^*a*^	47.1 ± 21.1	39.5 ± 24.8	16.9 ± 12.5	31.5 ± 18.3
*n*	*9*	*77*	*3*	*26*
ALSFRS-R score^*a*^	36.0 ± 5.1	39.5 ± 5.4	46.3 ± 1.5	34.7 ± 11.2
*n*	*8*	*51*	*4*	*22*

*AS, apparently sporadic; M/F, male/female; UL, upper limb; LL, lower limb; ALSFRS-R, amyotrophic lateral sclerosis rating scale-revised. ^*a*^Value are means ± SD. ^*b*^vs. familial cases: p < 0.0001.*

### Influence of the Generation and Heritability

The study of the 57 families showed that either ALS, ALS/FTD, or FTD could be noted in 65 relatives belonging to the former generation (parents, uncles, aunts) of the index cases; 8 grandparents were also affected as well as 41 siblings (Table 5). In ALS cases, clinical comparison showed a significantly earlier age of onset in index cases compared to the former generation (*p* = 0.005) but not with their siblings. Correlatively, siblings also had an earlier age of onset compared to the former generation (*p* = 0.027). Disease duration was similar between the groups. A clear and significant female predominance could be noted in the former generation (gender ratio 0.44) compared to the index cases (1.45, *p* = 0.011); no female predominance could be noted in the siblings. The group of ALS/FTD patients was a smaller group, but the same differences could be noted, with a male predominance in index cases and siblings, and an earlier onset, compared to their parents, but those differences were not significant. On the contrary, in all the groups of ALS/FTD patients, the upper limb onset was underrepresented. In the group of FTD, once again, we can note a female predominance in parents and grandparents, but not in sibs.

**TABLE 5:** Clinical characteristics according to parentality.

	n	Gender	Site onset	Age of onset^*a*^	Age at death^*a*^	Duration^*a*^
		(M/F)	(B/UL/LL/D)			
**ALS**						
Index cases	49	29/20^*b*^	17/10/21	55.2 ± 10.5(49)^*c*^	58.9 ± 10.0(44)	40.8 ± 24.8(44)
Grand parents	2	2/0	2/0/0	69.0±*na*(1)	65.0 ± 9.9(2)	36.0±*na*(1)
Parents	39	12/27	7/2/8	62.8 ± 10.4(23)	64.2 ± 11.4(20)	41.2 ± 25.0(22)
Siblings	28	14/14^*d*^	8/4/8	56.4 ± 8.1(21)^*e*^	60.5 ± 8.2(20)	38.2 ± 24.6(19)
**ALS/FTD**						
Index cases	21	13/8	8/6/7	60.6 ± 7.0(21)	63.4 ± 7.1(18)	29.7 ± 18.7(18)
Parents	6	2/4	3/2/1	62.5 ± 6.2(5)	65.0 ± 6.6(5)	31.0 ± 18.1(5)
Siblings	10	7/3	2/0/5/1	58.4 ± 7.8(8)	63.4 ± 6.8(7)	30.3 ± 20.0(6)
**FTD**						
Grand parents	6	2/4	–	58.0±*na*(1)	62.5 ± 3.5(2)	84.0±*na*(1)
Parents	20	9/11	–	64.8 ± 10.2(7)	74.0 ± 8.3(10)	88.1 ± 38.5(7)
Siblings	3	3/3	–	73.0 ± 1.7(2)	75.0±*na*(1)	

*M/F, male/female; B/UL/LL/D, Bulbar/Upper limb/Lower limb/Dementia. ^*a*^Value are means ± SD (n). ^*b*^Gender ratio vs. parents, p = 0.011. ^*c*^Age of onset vs. parents, p = 0.005. ^*d*^Gender ratio vs. parent, p = 0.027. ^*e*^Age of onset vs. parents, not significant.*

To refine the study of clinical characteristics between generations, we constituted two generational groups, the first group comprising index cases and their sibs affected of ALS or ALS/FTD (*n* = 108), thus corresponding to the same generation, and the second group composed of subjects from the parental generation (parents, uncles, and aunts, *n* = 44, Table 6). The comparison between these two groups showed a significant difference for gender (*p* = 0.004), age of onset (*p* = 0.003), and age at death (*p* = 0.007). There was no difference in the distribution of sites of onset nor for disease duration between the groups.

**TABLE 6:** Clinical characteristics of index cases + sibs and parents with ALS and ALS/FTD.

	Parents	Index cases + sibs	*p*
Gender: M/F (ratio)	14/30 (0.47)	63/45 (1.4)	0.004
Age of onset^*a*^	63.0 ± 9.7 (27)	56.9 ± 9.3 (99)	0.003
Bulbar onset (%)	43.5	35.5	
Upper limb onset (%)	8.7	20	ns
Lower limb onset (%)	43.5	42.5	
Dementia (%)	4.3	2	
Age at death^*a*^	64.7 ± 10.7 (24)	60.5 ± 8.9 (89)	0.007
ALS duration^*a*^	39.6 ± 24.3 (26)	37.2 ± 23.5 (87)	0.710

*M/F, male/female. ^*a*^Value are means ± SD (n).*

As the study of gender is potentially biased by an overrepresentation of women in some large families, we thus studied direct transmission of the trait by comparing pairs with mother-to-child transmission to those with father-to-child-transmission. There were 54 pairs with mother-to-child transmission and 33 with father-to-child, confirming the gender disequilibrium among parents. Comparison of the age of onset between parents and children from these pairs showed a significant difference with 62.7 ± 9.1 years in parents and 58.3 ± 8.5 years in children (*p* = 0.015). As an overrepresentation of women in pairs could also be due to their longer life span, and as penetrance of C9RE is age dependent, we also compared the age of onset of mothers and fathers from parent–child pairs. In women, the age of onset was 61.4 ± 11.3 years for ALS cases and 61.7 ± 8.1 years for FTD cases. In men, the mean age of onset was 59.6 ± 5.8 years for ALS cases and 72.2 ± 1.9 years for FTD cases. These differences were not significant.

### Inter- and Intrafamilial Correlations

To compare intra- and interfamilial phenotype similarities, the ICCs with their 95% CI were estimated. There was a significant intrafamilial correlation (more similarities among the members of the same family than between families) for the bulbar onset of ALS, with an ICC = 0.35 (95% CI: 0.004–0.717). For the upper limb onset and the lower limb onset, the ICCs were 0.13 (0; 0.670) and 0.009 (0; 0.670), respectively, with a 95%CI including 0, non-significant. For the age at onset, duration of ALS, or gender, ICCs were close to 0, and thus non-significant.

### Patients With Psychiatric Disorders

There were 13 patients with psychiatric disorders. Suicide was the most represented (*n* = 9), and other disorders included schizophrenia (in a nephew of the index case, *n* = 1), hypochondria (in a mother, *n* = 1), and bipolar disorder (in a son, *n* = 1). A last patient, father of an index case, was described as followed in a psychiatric hospital without any other detail. Suicide was noted in two fathers of index cases, one paternal uncle, three brothers, two sisters, and a nephew. Age at suicide could be ascertained for only six out of the nine relatives. The mean age at suicide was 54 years, and ages were 30, 52, 54, 60, 60, and 68 years. One patient who committed suicide was the father of an apparently sporadic ALS case; all the other belonged to the FALS group. In families, suicide was noted in almost all the family types, but was more frequent in families with only ALS cases (Table 1). C9RE could be studied in one patient with psychiatric disorder, the individual with schizophrenia, and the presence of a pathological expansion was confirmed. His father had died of ALS.

## Discussion

This study describes one of the largest cohorts, to date, of patients with C9RE and their familial clinical profile. Out of the 70 index cases described, 18.6% of the patients had no apparent familial history of ALS or FTD. This is larger than estimated in previous works; however, familial inquiry was missing in two cases, and pedigree was not informative in three other cases. In only three cases (4%), parents were old and alive without any neurological or psychiatric disorder (Majounie et al., 2012). In the 57 families identified, no clear predominance of a particular phenotype was noted. Similarly, psychiatric antecedents in relatives were noted in almost all the familial phenotypes with no particular predominance of suicides in families with FTD or ALS/FTD cases.

Compared to our database of sporadic ALS cases, the only difference in clinical characteristics was the earlier age of onset of C9RE ALS and ALS/FTD cases, and this has already been underlined (Byrne et al., 2012; Majounie et al., 2012; Cooper-Knock et al., 2014). Disease duration was not shorter compared to other series. Although it had been already suggested that patients with C9RE had more frequent bulbar onset, we did not find such a predominance neither for ALS nor ALS/FTD C9RE patients in the present cohort. Sporadic ALS patients from our database have not been tested for C9RE. However, only 3–7% of sporadic ALS patients may carry an abnormal C9RE. This low percentage suggests that, even if some of those patients are present in this sporadic ALS group, it would have only a marginal effect on population characteristics.

ALS and ALS/FTD C9RE patients did not differ statistically in any of the clinical criteria even though the gender ratio clearly favored predominance of men in the ALS/FTD group conversely to the ALS group. Disease duration was shorter in ALS/FTD patients, as already described, but the size of the samples was limited and the great variability of duration within groups potentially explains the absence of statistical difference. The study of clinical characteristics between apparently sporadic patients and familial cases was also limited by the size of the samples. Nevertheless, a significantly earlier age at onset was noted in the group of apparently sporadic patients with ALS. The shortest duration was recorded in the group with ALS/FTD, but it only comprised three patients and no further interpretation seems possible at this stage.

One important question when facing new gene abnormalities and particularly those with expansion is the existence of an anticipation. This is all the more important as the exact determination of C9RE cannot be routinely done and it is the reason why anticipation with C9RE has been scarcely studied, but the rare works on that topic were in favor of an anticipation (Van Mossevelde et al., 2017). In our cohort, comparison of clinical characteristics between generations showed substantial differences. The age of onset between index cases and siblings was similar and significantly earlier than for their parents, uncles, and aunts by 7 years in ALS and 2 years in ALS/FTD cases, thus suggesting anticipation. This was reinforced by the study of parent–child pairs. However, it is not possible to determine the exact cause of a possible anticipation. It may be due to changes in the size of C9RE, to other genetic aspects, to epigenetic or environmental factors. One other interesting difference between these groups is the female predominance in the parental generation that reached significance, with a gender ratio of 0.47 in parents vs. 1.4 in index cases (*p* = 0.004). Although a female predominance in C9RE carriers has already been shown, the gender disequilibrium we describe between generations has not been previously described, to our knowledge (Curtis et al., 2017). This may suggest that C9RE is different between men and women, as it has been demonstrated in other neurological disorders such as myotonic dystrophy or Huntington’s disease. The longer life span of women compared to men seems unlikely to explain such a gender disequilibrium as age of onset of parents with C9RE in the present cohort is not different between men and women.

It was interesting to compare the intrafamilial similarities of the phenotype to interfamilial ones. Bulbar onset was significantly less variable within families than between families suggesting that genetic aspects may underlie the occurrence of such onset in ALS. This was reinforced by the absence of intrafamilial correlation for gender. Indeed, as bulbar ALS patients are more frequently women (twice as more frequent than in men), this shows that gender was not a potential confounder for the intrafamilial similarity of bulbar onset between relatives.

Some but not all previous works have suggested that patients with C9RE may present with initial psychiatric symptoms. A higher rate of psychiatric disorders in ALS kindreds has also been described. The search for C9RE in individuals who died of suicide in Finland did not reveal any abnormal repeat expansion in the C9ORF72 region (Solje et al., 2016). In the present cohort, 9 patients had a suicide history in their family, and 4 other psychiatric disorders were found, for a total of 13, corresponding to 18.5% of index cases with a psychiatric disorder in their family and 12.8% with suicide. In France, the incidence of death by suicide is 11.8 per 100,000 people, and most of them occur between 45 and 64 years (Observatoire national du suicide, rapport 2014)^2^. While the mean age of suicide in our cohort is in the same range than in the general population, incidence of suicide in ALS families does not seem to be a chance association, all the more as the number of cases collected is likely to be underestimated. Indeed, familial information was obtained by a simple inquiry and a systematic medical ascertainment of causes of death may well have found more cases. Only one individual with psychiatric disorder could be genetically analyzed and carried C9RE. He was first diagnosed with schizophrenia at the age of 20. However, it is not possible to determine whether or not this disorder is a consequence of C9RE.

This study has some limitations. The cohort represents the recruitment of an ALS center, and it is possible that another source of recruitment, such as an FTD expert center for example, could have a somewhat significant clinical spectrum to describe. Moreover, we paid attention mainly to ALS cases, but FTD cases have not been studied neuropsychologically for subtype description. One other limit is that despite the size of the cohort, the limited number of subjects in subgroups precludes the analysis of all aspects of phenotypes with enough statistical power. Although we tried to ascertain diagnoses as much as possible, it was not possible for all the cases. Subsequently, it is not possible to exclude that some clinical phenotypes, not described in this cohort, may exist within ALS families. However, it seems unlikely that unusual phenotypes are frequent. Memory as well as knowledge biases may also underestimate some cases such as suicide for example, as this diagnosis may also be hidden within families. This is a descriptive and retrospective work without deep genetic analysis. It is also a center-based cohort, not a population-based study, and these points limit the scope of the conclusions. Subsequently, we cannot ascertain that genetic anticipation does exist even though the age of onset is significantly younger.

This cohort was composed of patients referred to our ALS center for suspicion of ALS. Among the 70 ALS index cases carrying C9RE, 57 familial cases were identified, with a total of 200 relatives with either ALS, ALS/FTD, FTD, or psychiatric disorder. No other neurological disorder was identified, making it unlikely that the pathological phenotype linked to C9RE is broad or frequent. Apparently, sporadic cases had an earlier age of onset. In familial cases, index cases and their siblings had an earlier age of onset compared to their parental generation, characterizing anticipation. In the parental generation, a significant female predominance was shown for which we have no formal explanation to date. A significant intrafamilial correlation of bulbar onset of ALS was also shown. In the families of index cases, a high incidence of suicide was described suggesting a direct link with C9RE, and physicians should be aware of that possibility. Studies on larger international cohorts with familial inquiry are warranted to confirm the existence of anticipation and to confirm and explain the reason for gender disequilibrium.

## Data Availability Statement

The raw data supporting the conclusions of this article will be made available by the authors, without undue reservation, to any qualified researcher.

## Ethics Statement

The studies involving human participants were reviewed and approved by CCPPRB, CHU Pitié-Salpêtrière, Paris, France. The patients/participants provided their written informed consent to participate in this study.

## Author Contributions

FE, RJ-M, NP, EB, EL, VD, and WC recruited patients and obtained patient consent. SA and LL were responsible for the databases. KM, AP, and SL were responsible for the genetics studied. M-CP, EN, and WC were responsible for statistical analyses. WC and FE drafted the manuscript. All authors critically reviewed the manuscript.

## Conflict of Interest

The authors declare that the research was conducted in the absence of any commercial or financial relationships that could be construed as a potential conflict of interest.

## Footnotes

1 https://www.xlstat.com
